# COX-2: A Pivotal Enzyme in Mucosal Protection and Resolution of Inflammation

**DOI:** 10.1100/tsw.2006.122

**Published:** 2006-05-25

**Authors:** John L. Wallace

**Affiliations:** Inflammation Research Network, Department of Pharmacology and Therapeutics, University of Calgary, Calgary, Alberta, T2N 4N1, Canada

**Keywords:** inflammation, cyclooxygenase, prostaglandin, lipoxin, colitis, colonic cancer, inflammatory bowel disease, irritable bowel syndrome

## Abstract

The discovery of a second form of cyclooxygenase, COX-2, led to a burst of research aimed at the development of nonsteroidal anti-inflammatory drugs that would not damage the gastrointestinal tract. In the years since, this promise has only been partially fulfilled. Selective COX-2 inhibitors cause less gastric damage than conventional, nonselective COX inhibitors, but their use is still associated with significant gastrointestinal injury, and with toxicity in the renal and cardiovascular systems. COX-2 is now recognized as a source of mediators that produce many beneficial and detrimental effects in the digestive system. In this review, the roles of COX-2 in mucosal defense and injury are discussed. Furthermore, contributions of COX–2—derived products to the long-term consequences of intestinal inflammation, including cancer, are reviewed.

Nonsteroidal anti-inflammatory drugs (NSAIDs) have been associated with gastrointestinal (GI) injury for a century. It was the discovery by Vane in 1971 that these drugs suppress the synthesis of prostaglandins (PGs) that first raised the notion that these autocoids may play a role in the maintenance of GI mucosal injury[1]. Shortly thereafter, PGs were shown to protect the stomach and intestine against injury induced by a variety of agents, including NSAIDs and ethanol[2]. PG analogues (e.g., misoprostol) were marketed as "gastroprotective" agents and had modest commercial success. The discovery of a second isoform of cyclooxygenase (COX), COX-2, in 1991[3] resulted in an enormous burst of activity among many pharmaceutical companies who recognized the potential utility of drugs that would selectively inhibit PG synthesis at sites of inflammation. The key underlying hypothesis for this strategy was that COX-2 was expressed solely at sites of inflammation, whereas COX-1 was constitutively expressed in tissues like the stomach and kidney, and produced the PGs responsible for "housekeeping" functions in these tissues[4]. Thus, it was proposed, selective inhibition of COX-2 would not produce the adverse effects seen with conventional NSAIDs in the GI tract or kidney, but would produce the same beneficial effects in terms of reducing inflammation and pain. This hypothesis was advanced very strongly by several pharmaceutical companies, and quickly became accepted by many as fact. However, several studies published since the mid-1990s demonstrated that the "COX-2 hypothesis" was a gross oversimplification[5,6].

As had been predicted, COX-2 was either undetectable or expressed at very low levels in the healthy GI tract of humans and various animals[7]. COX-1 is constitutively expressed throughout the GI tract and, at least in the absence of damage or inflammation, is the major source of PG synthesis in these tissues[8]. Gastric PG synthesis in COX-1–deficient mice was found to be negligible[9].

Gastric inflammation is very common in humans, as it is found invariably in people who have *Helicobacter pylori* colonizing their stomachs (~50% of the world's population)[10]. COX-2 is strongly expressed in inflamed gastric mucosa[11]. Moreover, COX-2 is rapidly expressed when the mucosa is damaged, and appears to play a very important role in facilitating ulcer repair[12,13,14,15]. Administration of selective COX-2 inhibitors to rodents with gastric ulcers results in retardation of the healing process[13,14,15]. However, administration of a selective COX-1 inhibitor or disruption of the COX-1 gene does not affect gastric ulcer healing[15].

One of the first observations suggesting that COX-2–derived PGs contribute to mucosal defense came from experiments performed on COX-1–deficient mice[9]. While these mice did not exhibit spontaneous gastric injury, despite negligible PG synthesis, administration of an NSAID did cause gastric damage. It is possible that this damage occurred as a consequence of topical irritant effects of the NSAID on the gastric mucosa[16], but another possibility is that COX-2–derived PG synthesis contributes importantly to mucosal defense, and its suppression by the NSAID therefore caused the gastric injury[8]. The latter hypothesis is supported by a number of findings. Maricic et al.[17] reported that induction of gastric ischemia-reperfusion in rats resulted in very little gastric damage. However, if the rats were pretreated with a selective COX-2 inhibitor, the damage was much worse. Intragastric instillation of hydrochloric acid has been found to cause marked up-regulation of COX-2, but not COX-1, in the stomach[18]. Administration of a selective COX-2 inhibitor during the period of exposure of the stomach to acid resulted in the formation of gastric erosions.

As mentioned above, the observation that an NSAID caused gastric damage in COX-1–deficient mice suggested that COX-2 contributed to gastric mucosal resistance to injury. However, this was somewhat paradoxical given that COX-2 expression had only been observed at very low levels in the stomach of healthy animals or humans[7]. We observed that, while the expression of COX-2 in the healthy stomach was very low, it could be very rapidly up-regulated. For example, COX-2 expression in the rat stomach was markedly increased within 1 h of oral administration of aspirin or indomethacin[19] (Fig. 1). The same has now been described to occur in the stomach and intestine after administration of selective COX-1 inhibitors[20]. As the induction of COX-2 could be prevented by administration of PGs, this up-regulation was likely occurring as a compensatory response to diminished mucosal PG levels[19,20].

These studies led us to more closely examine another tenet of the "COX-2 hypothesis"; namely, that suppression of COX-1 activity by NSAIDs accounts for the gastric damage caused by these agents. We tested this hypothesis by examining the effects of selective inhibition of COX-1 and COX-2 on gastric mucosal integrity in the rat[8]. Administration of a selective COX-2 inhibitor (celecoxib) did not cause significant gastric damage (Fig. 2). Administration of a selective COX-1 inhibitor (SC-560) also did not cause significant gastric damage. However, when a selective COX-1 inhibitor and a selective COX-2 inhibitor were both given to rats, gastric damage was elicited that was similar in severity to that seen after administering a conventional, nonselective NSAID (indomethacin). We also found that an NSAID with greater selectivity for COX-1 than COX-2, ketorolac, did not cause gastric damage when given at doses that caused near complete suppression of gastric PG synthesis, but did not inhibit COX-2. It was only when the dose of ketorolac was increased such that it significantly inhibited COX-2 that we began to see gastric erosion formation[8]. Mechanistic studies revealed that suppression of COX-1 accounted for the diminished gastric blood flow that is observed following NSAID administration, while suppression of COX-2 accounts for NSAID-induced leukocyte adherence to the vascular endothelium (Fig. 3).

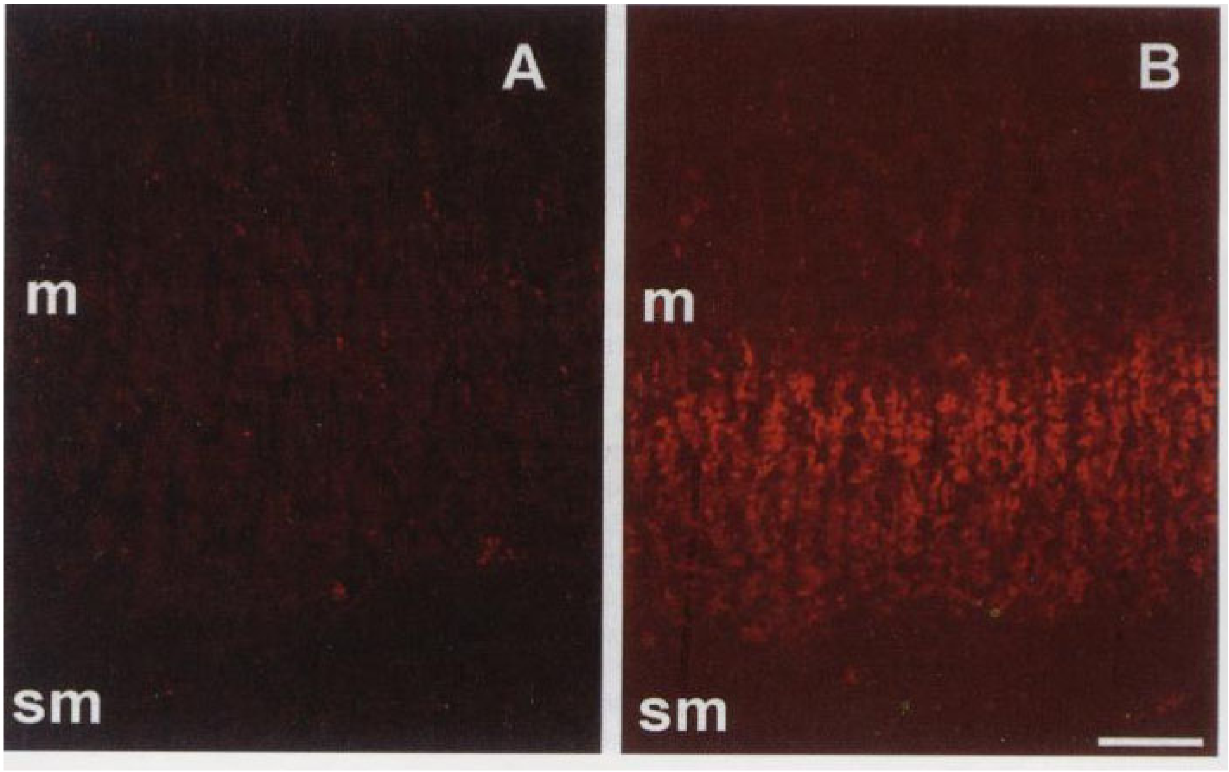

**FIGURE 1.** Expression of COX-2 in the rat stomach. Panel A shows COX-2 immunostaining in the normal stomach, while panel B shows the marked induction of COX-2 1 h after oral administration of aspirin (250 mg/kg). m, mucosa; sm, submucosa. The bar at the bottom of panel B represents 15 µm. Further details of these experiments can be found in Davies et al.[19].

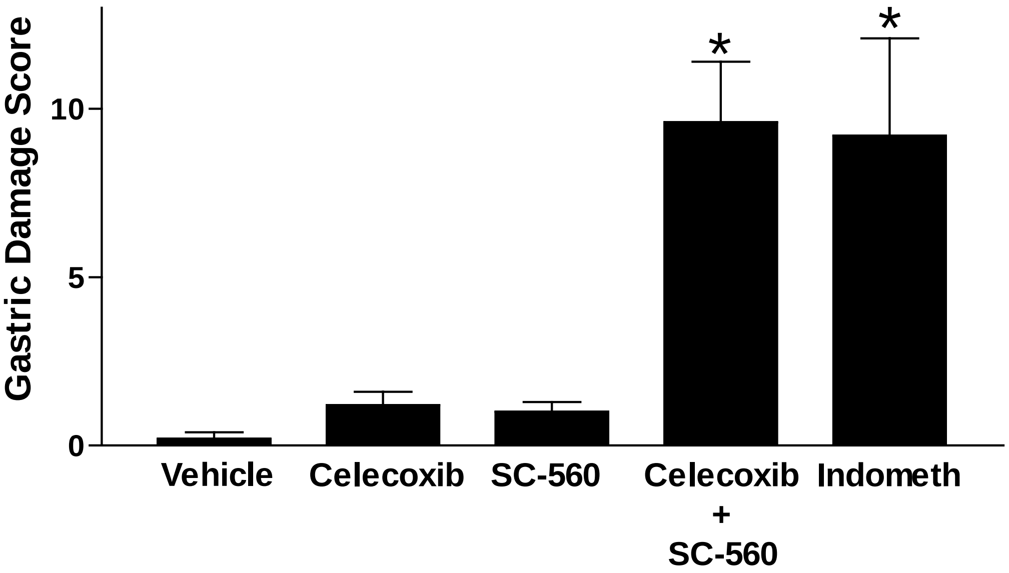

**FIGURE 2**. Combined inhibition of COX-1 and COX-2 is necessary for NSAID-induced gastric damage. Severity of gastric damage induced in the rat by administration of selective inhibitors of COX-2 (celecoxib; 15 mg/kg), COX-1 (SC-560; 40 mg/kg), or a nonselective COX inhibitor (indomethacin; 5 mg/kg). Asterisks indicate a significantly greater degree of damage than was observed with the vehicle ($p < 0.05$). Further details of these experiments can be found in Wallace et al.[8].

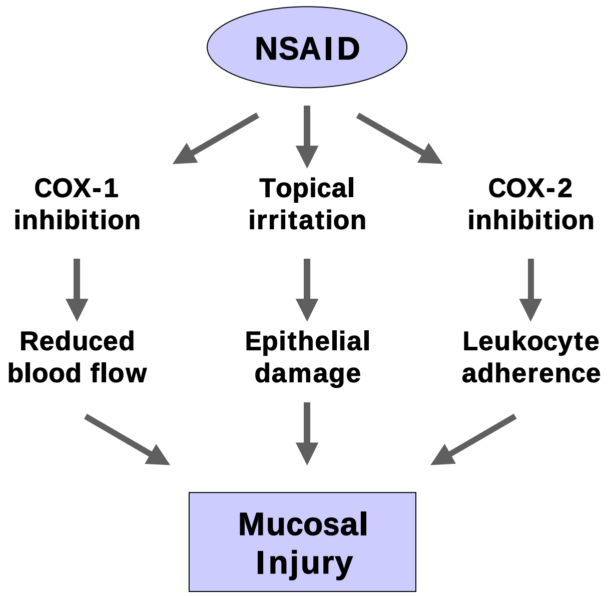

**FIGURE 3.** Pathogenesis of NSAID-induced gastric damage. Inhibition of COX-1 and COX-2, as well as topical irritant properties of NSAIDs, each contribute to the elicitation of gastric damage.

## COX-2 AND RESOLUTION OF INFLAMMATION

COX-2 was first regarded as a target for anti-inflammatory drugs. Suppression of the activity of this enzyme reduces edema formation and hyperalgesia. However, COX-2 is also a major contributor to the processes that lead to resolution of inflammation. A study of paw edema in COX-2–deficient and wild type mice underscored the importance of COX-2 in resolution of inflammation[21]. Injection of carrageenan into a hind paw resulted in marked edema formation over the hours that followed. The greatest increase in paw swelling was typically seen 4–6 h after carrageenan administration, and in wild type mice, the swelling subsided thereafter such that the paw was back to it normal volume by 24–48 h (Fig. 4). In COX-2–deficient mice, the extent of paw edema induced by carrageenan was similar to that in wild type mice. NSAIDs could reduce the paw swelling in the COX-2–deficient mice, indicating that COX-1-derived PGs contributed to the inflammatory response. More interesting, however, was the observation that the paw swelling in the COX-2–deficient mice failed to resolve even 1 week later. As well as exhibiting the same degree of swelling as during the acute response to carrageenan, the paw tissue exhibited significant leukocyte infiltration[21].

At around the same time, Gilroy and colleagues in London were examining the role of COX-2 in a rat model of carrageenan-induced pleurisy[22]. Carrageenan administration resulted, over a 24-h period, in significant leukocyte infiltration into the pleural cavity, and marked increases in $PGE_2$ synthesis. During the period 24–36 h after carrageenan administration, the numbers of leukocytes in the pleural cavity decreased to normal levels, indicative of resolution of the inflammatory response. Interestingly, it was precisely during this period that the greatest increase in COX-2 expression was observed. Accompanying the increased COX-2 expression was a marked increase in the levels within the pleural exudate of $PGD_2$. Administration of a selective COX-2 inhibitor abolished the $PGD_2$ production and prevented the decrease in leukocyte numbers in the pleural cavity. Gilroy et al.[22] went on to demonstrate that it was a hydration product of $PGD_2$, called 15-deoxy-$\Delta^{12-14}PGJ_2$, that was responsible for driving the resolution of the inflammatory response in the pleural cavity.

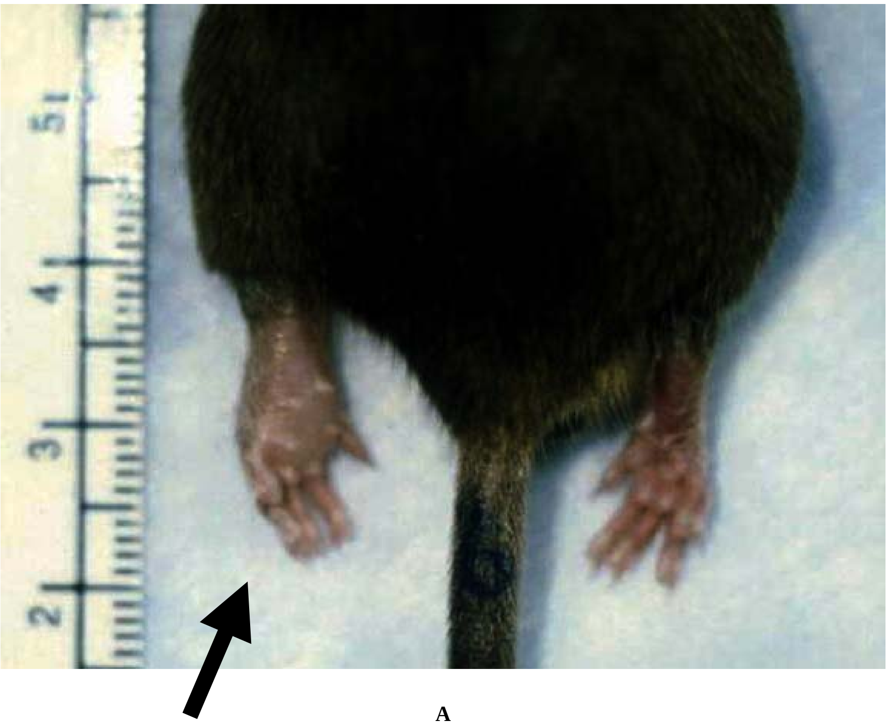

A

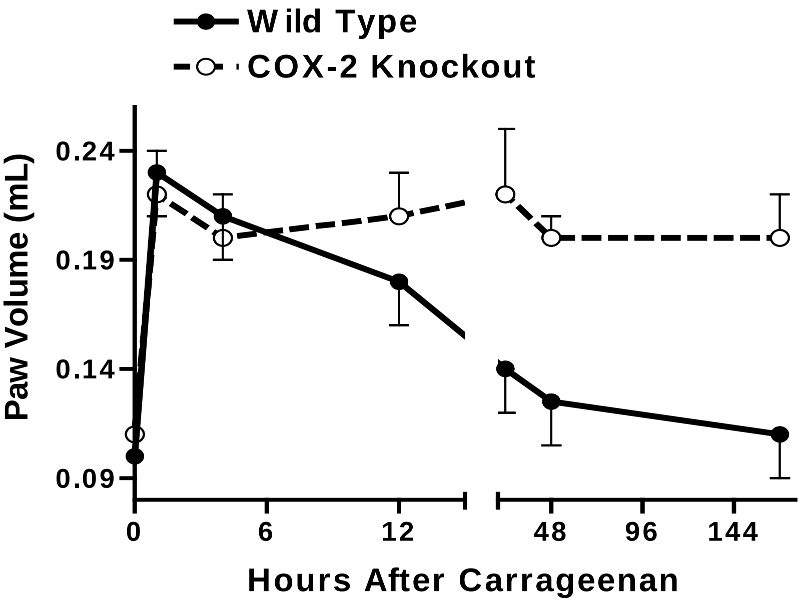

B

**FIGURE 4.** Carrageenan-induced inflammation of the paw in COX-2-deficient and wild type mice. Panel A shows the appearance of the paw of a mouse 1 week after subplantar injection of carrageenan (the paw on the left was injected). Note that the paw appears swollen compared to the noninjected paw. Panel B shows the change in paw volume in COX-2–deficient and wild type mice up to a week after carrageenan injection. While the edema resolves in the wild type mice, it is still apparent in the COX-2–deficient mice 1 week after the injection of carrageenan. Further details of these experiments can be found in Wallace et al.[21].

## COX-2–DERIVED LIPOXINS

The studies of Gilroy et al.[22] identified COX-2 as a source of an important anti-inflammatory prostanoid. Studies of the effects of COX-2 inhibitors in the stomach revealed protective effects of another COX-2–derived anti-inflammatory substance. When there was first an indication that selective COX-2 inhibitors might have prothrombotic actions[23], some suggested that patients using these drugs should consider also taking low-dose aspirin in order to provide cardioprotection. Consideration was not given to the possibility that this would result in an increase in the risk of GI complications. Animal studies clearly demonstrated that the combined administration of low-dose aspirin and a selective COX-2 inhibitor did indeed result in significantly more gastric damage than was produced by either drug alone[24]. Moreover, clinical data confirmed this detrimental interaction[25,26]. Was the increased damage a consequence simply of the fact that a drug inhibiting COX-1 and a drug inhibiting COX-2 were together producing more complete inhibition of gastric PG synthesis? Possibly, but there was also another explanation. Studies performed by the group of Serhan had identified a novel group of lipid mediators, lipoxins (LX), which play important roles in the resolution of inflammation[27,28,29]. Lipoxins can inhibit various neutrophil functions[30] and can induce phagocytosis of apoptotic neutrophils by macrophages[31]. Interestingly, one particular lipoxin (15-R-epi-$LXA_4$) can be synthesized via a COX-2–dependent pathway[27,28,29]. Aspirin inhibits prostanoid synthesis by acetylating a key serine residue near the active site of COX-1 and COX-2. This results in a conformational change in the enzyme, which greatly limits the ability of arachidonic acid, the precursor of prostanoids, to gain access to the active site. Thus, prostanoid synthesis is irreversibly inhibited. In the case of COX-2, however, metabolism of arachidonic acid is not completely inhibited. While prostanoid synthesis is impaired following aspirin-acetylation of COX-2, the acetylated enzyme can convert arachidonic acid into 15-R-HETE (15-R-hydroxyeicosatetraenoic acid), which can subsequently be metabolized via 5-lipoxygenase to 15-R-epi-$LXA_4$[27].

We had observed many years ago that NSAIDs trigger leukocyte adherence in the GI microcirculation[32,33], and that this contributed significantly to mucosal injury[34,35,36,37]. If NSAID-induced leukocyte adherence was blocked, such as through administration of monoclonal antibodies against various adhesion molecules, the injury to the stomach was prevented[35,36]. Given that lipoxins are potent inhibitors of leukocyte adherence and are produced following aspirin administration[27], we postulated that aspirin-triggered lipoxin (ATL) might have protective effects in the stomach[24]. First, we measured lipoxin synthesis by the stomach in the absence and presence of aspirin. As had been reported for other tissues, aspirin did indeed trigger lipoxin synthesis by the stomach. Coadministration of a selective COX-2 inhibitor with aspirin suppressed the gastric lipoxin synthesis and resulted in a marked increase in the extent of gastric mucosal damage[24]. Administration of synthetic $LXA_4$ prior to aspirin resulted in a dose-dependent reduction in the extent of gastric damage. Thus, ATL acts to diminish injury to the gastric mucosa. Coadministration of a selective COX-2 inhibitor with aspirin blocks the formation of ATL, thereby resulting in more extensive gastric damage[38].

When the stomach is inflamed, $LXA_4$ may make an even greater contribution to mucosal defense. Induction of gastritis in the rat was associated with a significantly greater level of ATL synthesis, and greater resistance of the stomach to injury[39]. Pretreatment with a selective COX-2 inhibitor blocked the lipoxin synthesis and increased the susceptibility of the stomach to injury.

## COX-2 IN RESOLUTION OF COLITIS

Colitis can be induced in rats or mice through intracolonic administration of a hapten, such as trinitrobenzene sulfonic acid (TNBS)[40,41]. In the rat, there is a rapid onset of granulocyte infiltration into the mucosa, peaking at 72 h post-TNBS[41]. Epithelial damage is extensive and, over the course of the following week, the inflammation becomes transmural and chronic in nature (extensive lymphocyte and macrophage infiltration). COX-2 expression is markedly elevated in the mucosa within hours of

TNBS administration[42]. Accompanying this increase is an increase in the synthesis of $PGD_2$[43]. As was the case in the studies of Gilroy et al.[22], COX-2–derived $PGD_2$ acts as a “stop signal”, reducing granulocyte infiltration. Thus, selective COX-2 inhibition during the acute phase of TNBS-induced colitis results in a significant elevation of granulocyte infiltration[43]. Interestingly, during the second week after TNBS administration, when the colitis begins to resolve and the epithelium becomes reconstituted, mucosal $PGE_2$ synthesis returns to normal levels, but there is a sustained increase in $PGD_2$ synthesis[44]. Indeed, we observed elevated colonic $PGD_2$ synthesis and COX-2 expression for more than 6 weeks after TNBS administration, even though the colonic mucosa was no longer inflamed[44,45,46] (Fig. 5). Nevertheless, there were prolonged changes in mucosal structure and function, which appear in part to be related to the enhanced synthesis of $PGD_2$[44,47].

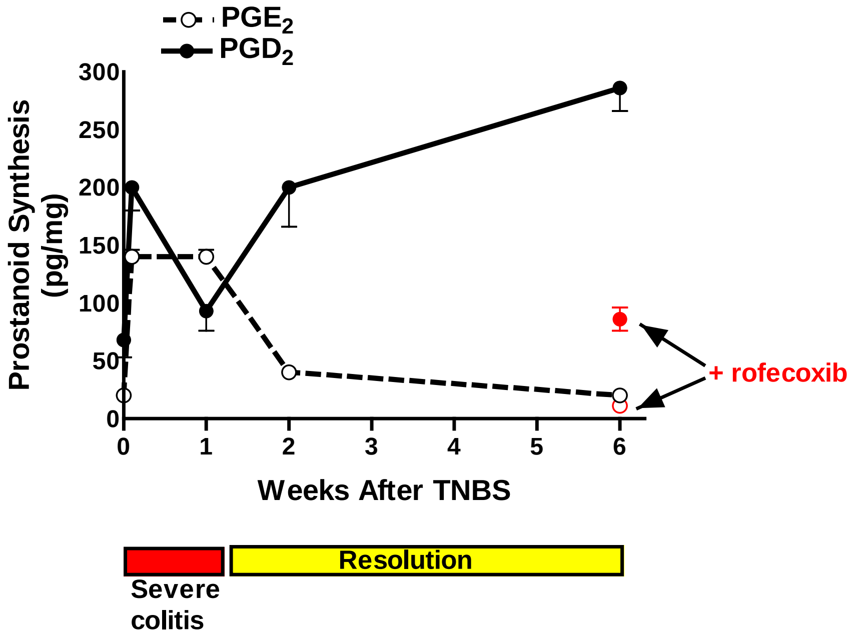

**FIGURE 5**. Colonic prostaglandin $E_2$ and $D_2$ synthesis during experimental colitis and during the period of resolution of the colitis. Note that $PGE_2$ synthesis returns to basal levels by 2 weeks after administration of TNBS, the agent that induces the colonic inflammation. However, $PGD_2$ remains markedly elevated at 6 weeks post-TNBS, when the inflammation has resolved. The $PGD_2$ synthesis can be inhibited by rofecoxib, indicating that it is derived from COX-2. Further details of these experiments can be found in Zamuner et al.[44].

The intestinal epithelium acts as a protective barrier, separating the contents of the lumen from the underlying lamina propria. Impairment of epithelial secretion and epithelial barrier function occurs during acute intestinal inflammation[48,49,50]. This dysfunction, which includes impaired epithelial secretion and elevated bacterial translocation, persists for weeks after resolution of the inflammatory response[44,45,46,47]. If similar changes occur in the intestine of humans following bouts of colitis, they may contribute to relapses of colitis and/or to generation of symptoms that characterize disorders such as irritable bowel syndrome. The elevated COX-2–derived $PGD_2$ synthesis in the colon of rats that we observed following resolution of TNBS-induced colitis appears to contribute to the prolonged alterations in epithelial function[44]. Thus, selective inhibition of COX-2 with rofecoxib reduced colonic $PGD_2$ synthesis to normal levels, and reversed the alterations in epithelial secretion and bacterial translocation[44].

There have been numerous case reports indicating that NSAIDs could exacerbate inflammatory bowel disease or cause its reactivation when NSAIDs were taken by a patient in remission[51]. Studies in the TNBS model confirmed the ability of NSAIDs to exacerbate colitis[52]. There is now convincing evidence from animal studies[42,53] that it is the inhibition of COX-2 by NSAIDs that is responsible for this effect. Clinical data consistent with this notion have also been reported[54].

## COX-2 IN PROGRESSION FROM COLITIS TO CANCER

Prostaglandins have long been suggested to contribute to tumor growth in various tissues. This notion was largely based on observations that selective COX-2 inhibitors and nonselective NSAIDS appear to reduce the incidence of colon cancer in humans and in experimental models[55,56]. The risk for colorectal cancer is increased in association with the extent and duration of inflammatory bowel disease[57,58,59]. Studies of animal models of colitis have demonstrated a similar increase in the risk of experimental colon cancer[60,61].

In addition to changes in colonic epithelial and barrier function in rats that had recovered from colitis, we observed a marked increase in mucosal thickness. We speculated that this increased proliferation of the epithelium could be a precursor of neoplastic changes in the colon. Clinically, it is well established that individuals with ulcerative colitis have an elevated predisposition to development of colon cancer. We further speculated that the increased COX-2 expression (and $PGD_2$ synthesis) that persists after resolution of colitis in the rat might contribute to the observed hyperproliferation, and to a predisposition to neoplastic changes in the colon. To test this hypothesis, we used a chemical model of precancerous lesion formation to determine first whether or not postcolitis rats exhibited an increased susceptibility to colon cancer. Healthy or postcolitis rats were given a carcinogen (azoxymethane) and several weeks later, we examined the colon for aberrant crypt foci (ACF). ACF are a well-established marker of the early stages of colon cancer development in rodents and humans[62,63]. They consist of dysplastic or hyperplastic crypts, and subsequent expansion generates larger adenomas, which in turn may proceed to carcinoma[64].

As suspected, azoxymethane admininstraion to rats that had recovered from a bout of colitis developed significantly more ACF than healthy rats (Fig. 6). We then examined the potential contribution of COX-2 and $PGD_2$ to the predisposition of postcolitis rats to ACF formation. Daily treatment with a selective COX-2 inhibitor for a week (prior to azoxymethane administration) reduced the number of ACF in postcolitis rats to the same levels as seen in healthy controls receiving the carcinogen. Daily treatment for a week with a $PGD_2$ (DP1) receptor antagonist (BW A868C) produced a similar effect (Fig. 6). Treatment with either of these agents also significantly reduced the mucosal hyperproliferation in the postcolitis rats. Thus, $PGD_2$ synthesis via COX-2 appears to make an important contribution to the postcolitis hyperproliferation of epithelial cells, and to the predisposition to neoplastic changes.

These studies identify COX-2 and $PGD_2$ as potential therapeutic targets for chemoprevention of colon cancer. However, given the important roles of COX-2 (in part through synthesis of $PGD_2$) in the resolution of healing and promotion of healing in the context of colitis[42,43,54], inhibition of COX-2 may not be a viable approach to reducing the risk of colorectal cancer in colitis patients. On the other hand, inhibition or down-regulation of downstream targets, such as prostaglandin D synthase and DP receptors, may be viable in this patient population.

## FUTURE DIRECTIONS

It is ironic that the development of a new class of anti-inflammatory drugs that were proposed to spare the GI tract of injury (i.e., selective COX-2 inhibitors) has resulted in a greatly improved understanding of the role of the target of those drugs in prevention and healing of GI injury. As the studies described above suggest, COX-2 plays key roles in the ability of the GI mucosa to respond to injury. Inhibition of COX-2

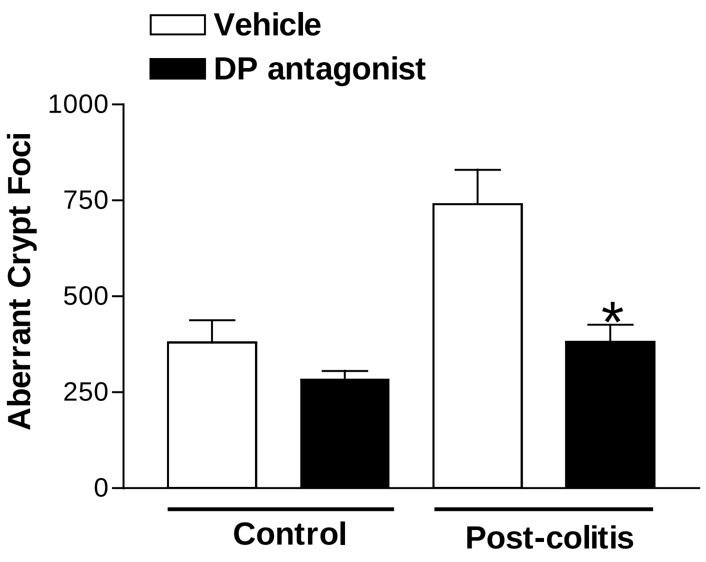

**FIGURE 6.** Colitis-associated preneoplastic lesions in the colon (ACF) are prevented by treatment for 1 week with a $PGD_2$ receptor antagonist (BW A868c). Note that postcolitis rats develop more ACF than control (healthy) rats given the same carcinogen. $*p < 0.05$ vs. the corresponding vehicle-treated group. Further details of these experiments can be found in Zamuner et al.[47].

activity in the face of injury results in an exacerbation of the injury and impairment of repair processes. Prolonged elevation of COX-2 expression appears to contribute significantly to long-term changes in GI function and proliferation. More research into the downstream products of COX-2 and their cellular and molecular targets will greatly aid in the development of novel therapies for chronic GI disorders, and for chemoprevention of GI cancer.

## ACKNOWLEDGMENTS

Dr. Wallace's research is supported by grants from the Canadian Institutes of Health Research and the Crohn's and Colitis Foundation of Canada. Dr. Wallace also receives salary support from the Alberta Heritage Foundation for Medical Research and from the Canada Research Chairs program.

## BIOSKETCH

**John Wallace** was born and raised in Toronto, Canada. He did BSc and MSc degrees at Queen's University, in Kingston, Ontario, then a PhD at the University of Toronto. That was followed by 3 years of post-doctoral studies in the Department of Mediator Pharmacology at Wellcome Research Laboratories in London, England. That work was carried out in the group headed by Brendan Whittle, Salvador Moncada and Nobel Laureate Sir John Vane. Dr. Wallace then returned to Canada as an Assistant Professor at Queen's University and in 1989 moved to the University of Calgary. He is now a Professor of Pharmacology & Therapeutics, a Fellow of the British Pharmacological Society, a member of the Brazilian Academy of Science, an Alberta Heritage Foundation for Medical Research Senior Scientist and holds a Canada Research Chair in Inflammation Research. Dr. Wallace is presently pursuing an MBA degree from the University of Birmingham (England).
